# Supplementary material for: Assessing equity of the spatial distribution of primary health care facilities in Fuzhou City, China: A comprehensive method
Source: PLoS One. 2021 Dec 28;16(12):e0261256. doi: 10.1371/journal.pone.0261256 (PMC8714018; doi:10.1371/journal.pone.0261256)
Supplement: Attachment — Submitted filename: Response to Reviewers.docx [file pone.0261256.s001.docx]

**Response to Reviewers**

**Journal**: PLOS ONE

**Title of the Article**: Assessing equity of the spatial distribution of primary health care facilities in Fuzhou City, China: A comprehensive method

**Manuscript Number**: PONE-D-21-20230

**Response**: We appreciate the time and efforts by the editor and reviewers in reviewing this manuscript. We have made modifications according to the comments of the editor and reviewers, and carried out the corrections one by one as suggested by the reviewers. All the changes have been highlighted in red in the revised manuscript. We hope that the revised version can meet the requirements of journal publication.

**Reviewers' comments:**

(1) The paper itself is well structured and the study well conducted. I agree that this study would gain a lot if the impact of the service ability of primary health care facilities on the equity assessment were taken into account.

**Response**: Firstly, we appreciate your recognition of my research work and manuscript writing. Secondly, for the comment “I agree that this study would gain a lot if the impact of the service ability of primary health care facilities on the equity assessment were taken into account”, I have considered this and mentioned it as “limitations” in the last paragraph at the end of the manuscript. At this stage, the impact of the service ability of primary health care facilities on the equity assessment was not taken into account due to the lack of relevant data. We will take it as one of the important directions and optimize it in future research. Thank you very much for your constructive suggestions.

**Journal requirements:**

(1) Please ensure that your manuscript meets PLOS ONE's style requirements, including those for file naming.

**Response**: Thank you very much, we have revised the styles of the manuscript body and title page according to the PLOS ONE style templates that you provided.

(2) Please ensure that you include a title page within your main document.

**Response**: Thank you very much, we have added the title page into the beginning of my manuscript.

(3) We note that Figure 1, 3, 4, 5 and 7 in your submission contain [map/satellite] images which may be copyrighted.

**Response**: Thank you very much, we have removed figures 1, 3, 4, 5 and 7 in the manuscript because it is difficult to obtain permission from the original copyright holder, and it has no great influence on the content expression of the manuscript. The changes of the corresponding part in the content expression have been highlighted in red in the revised manuscript.

(4) Please review your reference list to ensure that it is complete and correct.

**Response**: Thank you very much, we have reviewed my reference list and all the changes have been highlighted in red.
