# Supplementary material for: Assessing equity of the spatial distribution of primary health care facilities in Fuzhou City, China: A comprehensive method
Source: PLoS One. 2021 Dec 28;16(12):e0261256. doi: 10.1371/journal.pone.0261256 (PMC8714018; doi:10.1371/journal.pone.0261256)
Supplement: Attachment — Submitted filename: Response to Reviewers.docx [file pone.0261256.s002.docx]

**Response to Reviewers of the Second Review**

Dear Editor:

We appreciate the time and efforts of the editor and reviewers in the second review process of this manuscript. We have made responses for the comments of the editor, reviewers, and journal one by one. All the changes have been highlighted in red in the revised manuscript. We hope that the revised version can fully meet the requirements of journal publication.

**Response to Reviewers' Comments:**

***Comments:***

*1. If the authors have adequately addressed your comments raised in a previous round of review and you feel that this manuscript is now acceptable for publication, you may indicate that here to bypass the “Comments to the Author” section, enter your conflict of interest statement in the “Confidential to Editor” section, and submit your "Accept" recommendation.*

***Reviewer #1: All comments have been addressed***

*2. Is the manuscript technically sound, and do the data support the conclusions?*

***Reviewer #1: Yes***

*3. Has the statistical analysis been performed appropriately and rigorously?*

***Reviewer #1: Yes***

*4. Have the authors made all data underlying the findings in their manuscript fully available?*

***Reviewer #1: Yes***

*5. Is the manuscript presented in an intelligible fashion and written in standard English?*

***Reviewer #1: Yes***

*6. Review Comments to the Author*

***Reviewer #1: (No Response)***

**Response**: Thank you very much for your approval of my responses to your comments of the first review, and thank you for your recognition of my research work and other aspects as well.

**Response to Additional Editor Comments:**
***Comments:*** *Considering the reviewers suggestion, I am going with a decision of minor revision.*

**Response**: Thank you very much for your review of my manuscript, I have carefully revised it and made it meet PLOS ONE’s publication criteria.

**Response to Journal Requirements:**

***Requirements****:*

*Please review your reference list to ensure that it is complete and correct. If you have cited papers that have been retracted, please include the rationale for doing so in the manuscript text, or remove these references and replace them with relevant current references. Any changes to the reference list should be mentioned in the rebuttal letter that accompanies your revised manuscript. If you need to cite a retracted article, indicate the article’s retracted status in the References list and also include a citation and full reference for the retraction notice.*

**Response**: Thank you very much, we have reviewed my reference list again. Three references were modified and the changes have been highlighted in red in the “Revised Manuscript with Track Changes”. They are as follows:

(1) 4. Farhang L, Bhatia R, Scully CC, Corburn J, Gaydos M, Malekafzali S. Creating tools for healthy development: Case study of San Francisco’s eastern neighborhoods community health impact assessment. J Public Health Man 2008; 14(3):255-65. doi: 10.1097/01.PHH.0000316484.72759.7b.

(2) 39. Liu HX, Remme RP, Hamel P, Nong HF, Ren H. Supply and demand assessment of urban recreation service and its implication for greenspace planning: A case study on Guangzhou. Landsc Urban Plan 2020; 203:103898. doi: 10.1016/j.landurbplan.2020.103898.

(3) 40. Lee G, Hong I. Measuring spatial accessibility in the context of spatial disparity between demand and supply of urban park service. Landsc Urban Plan 2013; 119:85-90. doi: 10.1016/j.landurbplan.2013.07.001.
